# Supplementary figures and images for: Comprehensive Characterization of the C3HC4 RING Finger Gene Family in Potato (Solanum tuberosum L.): Insights into Their Involvement in Anthocyanin Biosynthesis
Source: Int J Mol Sci. 2024 Feb 8;25(4):2082. doi: 10.3390/ijms25042082 (PMC10889778; doi:10.3390/ijms25042082)

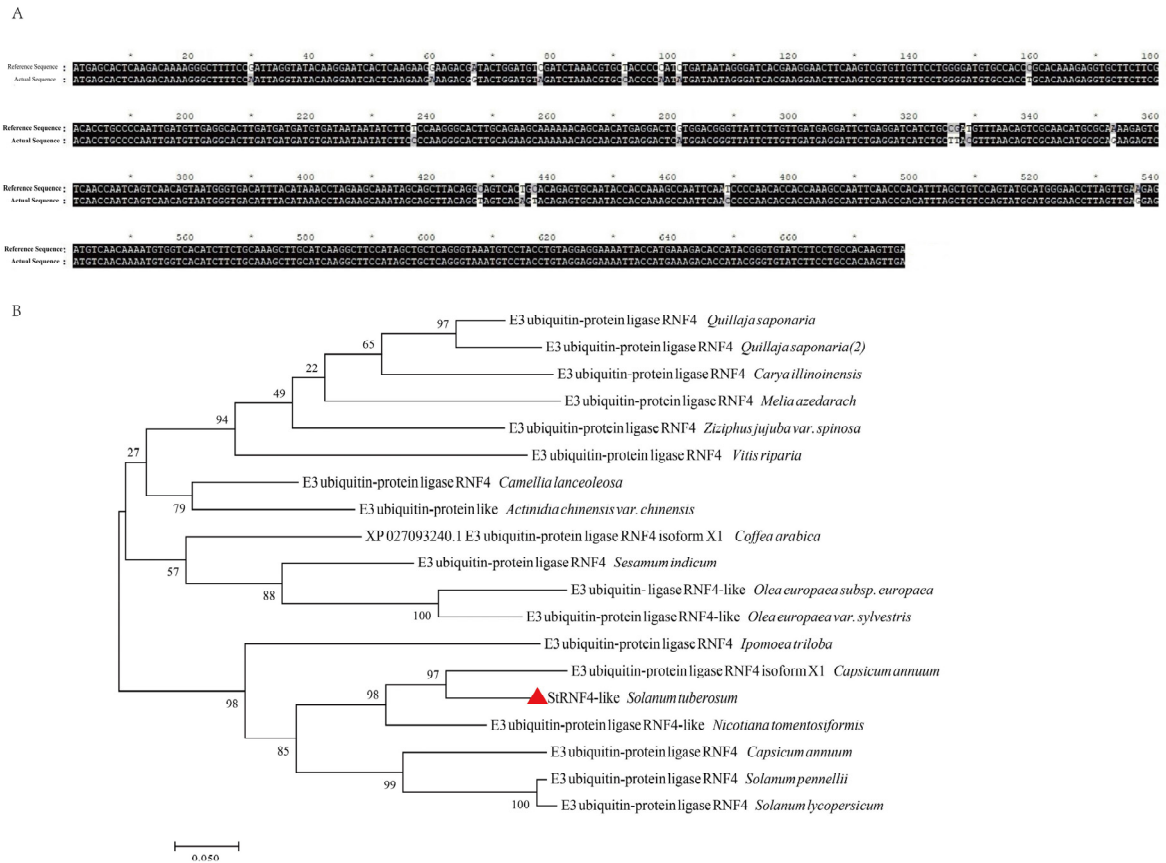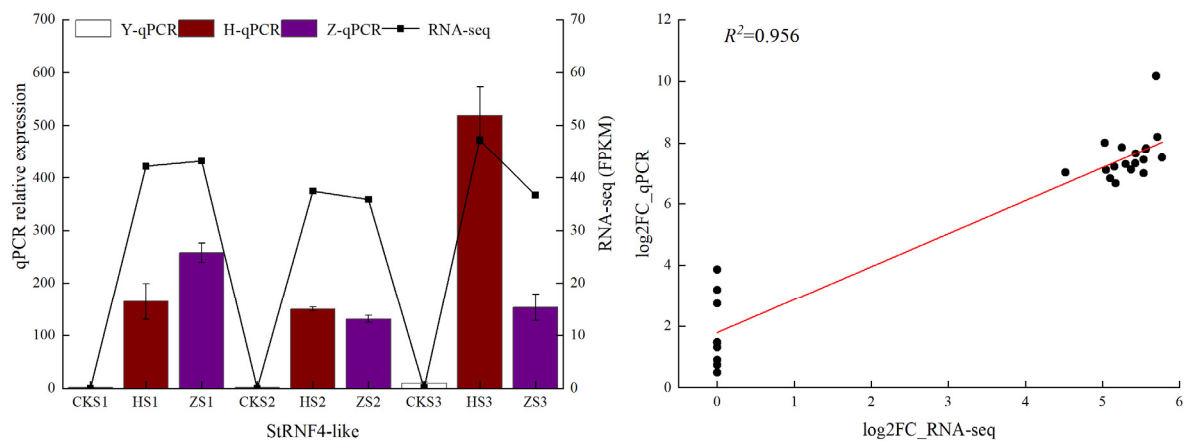

Supplement: Supplementary file 1 [file ijms-25-02082-s001.zip › supplementary figures.pdf]
